# Using CRISPR/Cas9-mediated gene editing to further explore growth and trade-off effects in myostatin-mutated F4 medaka (*Oryzias latipes*)

**DOI:** 10.1038/s41598-017-09966-9

**Published:** 2017-09-12

**Authors:** Ying-Chun Yeh, Masato Kinoshita, Tze Hann Ng, Yu-Hsuan Chang, Shun Maekawa, Yi-An Chiang, Takashi Aoki, Han-Ching Wang

**Affiliations:** 1 Institute of Biotechnology, College of Bioscience and Biotechnology, National Cheng Kung University, Tainan, 701 Taiwan; 2 Division of Applied Bioscience, Graduate School of Agriculture, Kyoto University, Kyoto, 606-8502 Japan; 3 Department of Biotechnology and Bioindustry Sciences, National Cheng Kung University, Tainan, Taiwan

## Abstract

Myostatin (MSTN) suppresses skeletal muscle development and growth in mammals, but its role in fish is less well understood. Here we used CRISPR/Cas9 to mutate the MSTN gene in medaka (*Oryzias latipes*) and evaluate subsequent growth performance. We produced mutant F0 fish that carried different frameshifts in the *Ol*MSTN coding sequence and confirmed the heritability of the mutant genotypes to the F1 generation. Two F1 fish with the same heterozygous frame-shifted genomic mutations (a 22 bp insertion in one allele; a 32 bp insertion in the other) were then crossbred to produce subsequent generations (F2~F5). Body length and weight of the MSTN^−/−^ F4 medaka were significantly higher than in the wild type fish, and muscle fiber density in the inner and outer compartments of the epaxial muscles was decreased, suggesting that MSTN null mutation induces muscle hypertrophy. From 3~4 weeks post hatching (wph), the expression of three major myogenic related factors (MRFs), MyoD, Myf5 and Myogenin, was also significantly upregulated. Some medaka had a spinal deformity, and we also observed a trade-off between growth and immunity in MSTN^−/−^ F4 medaka. Reproduction was unimpaired in the fast-growth phenotypes.

## Introduction

Myostatin (MSTN) is a member of the transforming growth factor-β (TGF-β) superfamily and is a well-known negative regulator of myogenesis in skeletal muscle development^1–5^. After MSTN is produced as a prepropeptide, a proteolytic process releases the C-terminal active domain of MSTN, which then dimerizes to become an activated peptide with biological activity^6–9^. The dimer of the MSTN C-terminal active domain subsequently interacts with membrane receptors and activates Smad2/3 signaling, which in turn initiates a signaling cascade that inhibits the expression of the myogenic regulatory factors (MRFs), including Myf5, myogenin and MyoD^10–12^. Double muscling (i.e. extreme gains in muscle mass) in MSTN-null phenotypes has been characterized in mammals^13–15^. This increase of muscle size in MSTN-null mammals may be caused by hypertrophy (increased muscle fiber size) or hyperplasia (increased number of muscle fibers) or both, and seems to be dependent on the animal species as well as the method used to suppress MSTN^16, 17^.

Fast-growing animals are usually preferred in commercial animal culture, and the effect of MSTN on fish first began to be studied nearly twenty years ago^13^. In fish, MSTN proteins are expressed ubiquitously in various tissues and organs, including those in the ingestion system and the reproductive system^18–20^. Most fish species carry two or more copies of MSTN. In teleosts, this has led to a diversification in MSTN functionality and/or mechanisms, and has given rise to various phenotypes in which changes in growth performance and skeletal muscle development can occur via both hyperplasia and/or hypertrophy^21–25^. This diversity means that there is no universal fish research model for MSTN. In the present study, we chose to investigate MSTN function in medaka (*Oryzias latipes*; rice fish). In evolutionary terms, medaka is separated from zebrafish by ~150 MY^26^. It is also unusual- and very convenient for our purposes- in that it has only a single copy of the MSTN gene^27^.

Methods that have previously been applied to generate MSTN-mutated medaka include transgenesis^24^, TILLING (targeting induced local lesions in the genome) by EMU-mutagenesis^27^, and TALENs (Transcription activator-like effector nucleases)^22^. Here, instead of temporary suppression by RNAi, or disabling MSTN by transgenesis or chemical mutagenesis, we used the CRISPR/Cas9 system to create several generations of MSTN^−/−^ medaka. CRISPR/Cas9 has recently been developed into a powerful endonuclease-based gene editing tool that can be used to mutate specific genes in both eukaryotes and prokaryotes^28–31^, and we anticipated that it would provide several advantages over the currently available alternative platforms. In TALENs, for example, the design and construction of the specific DNA binding protein is more complicated, the overall process is more expensive, and the platform’s mutagenic efficiency is lower. Meanwhile, TILLING induces only an in-frame point mutation instead of frame shifts that cause the deletion of an entire functional domain. On the other hand, in the CRISPR/Cas9 platform, the sgRNA can tolerate up to five mismatches, so it is necessary to ensure that no disruptive off-target effects are being induced at other genetic loci. In the first half of this study, after describing how we used the CRIPSR/Cas9 platform to produce MSTN^−/−^ medaka, we confirm that all of the fish carried frame-shifted mutations and that there were no off-target effects. In the second half, we examine the growth performance of the knock-out fish in terms of body weight, body length and the development of muscle fibers. We also monitor the expression of MRFs (myogenic related factors). In addition, since there is often a trade-off between growth and other biological traits, such as immunity^32^ and reproduction^33^, we evaluate virus susceptibility by challenging the MSTN^−/−^ medaka with RGNNV (red spotted grouper nervous necrosis virus) and assess their reproductive performance by monitoring both the number of eggs produced and the number of eggs that were successfully fertilized.

## Results

### Introduction of CRISPR/Cas9 mutations into the MSTN gene of medaka

**Figure 1:**
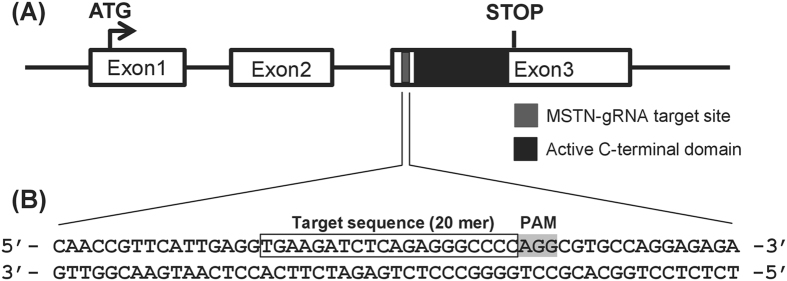
Our MSTN-sgRNA targets a location upstream of the active C-terminal domain of *Ol*MSTN. (**A**) Schematic representation of the *Ol*MSTN gene, which contains three exons, exon 1~3. The active C-terminal domain (black box) is found in exon 3. (**B**) The MSTN-sgRNA was designed to target a sequence (gray box) upstream of the active C-terminal domain in exon 3. The PAM sequence is shaded.

The medaka genome contains only a single ortholog of the mammalian MSTN (*Ol*MSTN; Ensembl gene no. ENSORLG00000015057), and we therefore designed our MSTN-sgRNA to specifically target this gene. As shown in Fig. 1, the active C terminal domain of MSTN is located in exon 3, and the MSTN-sgRNA target sequence was located upstream of this active domain on the 5′ end of the same exon.

### Breeding the F2-F5 MSTN^−/−^ medaka progeny and molecular characterization of their CRISPR/Cas9-mediated mutations

**Figure 2:**
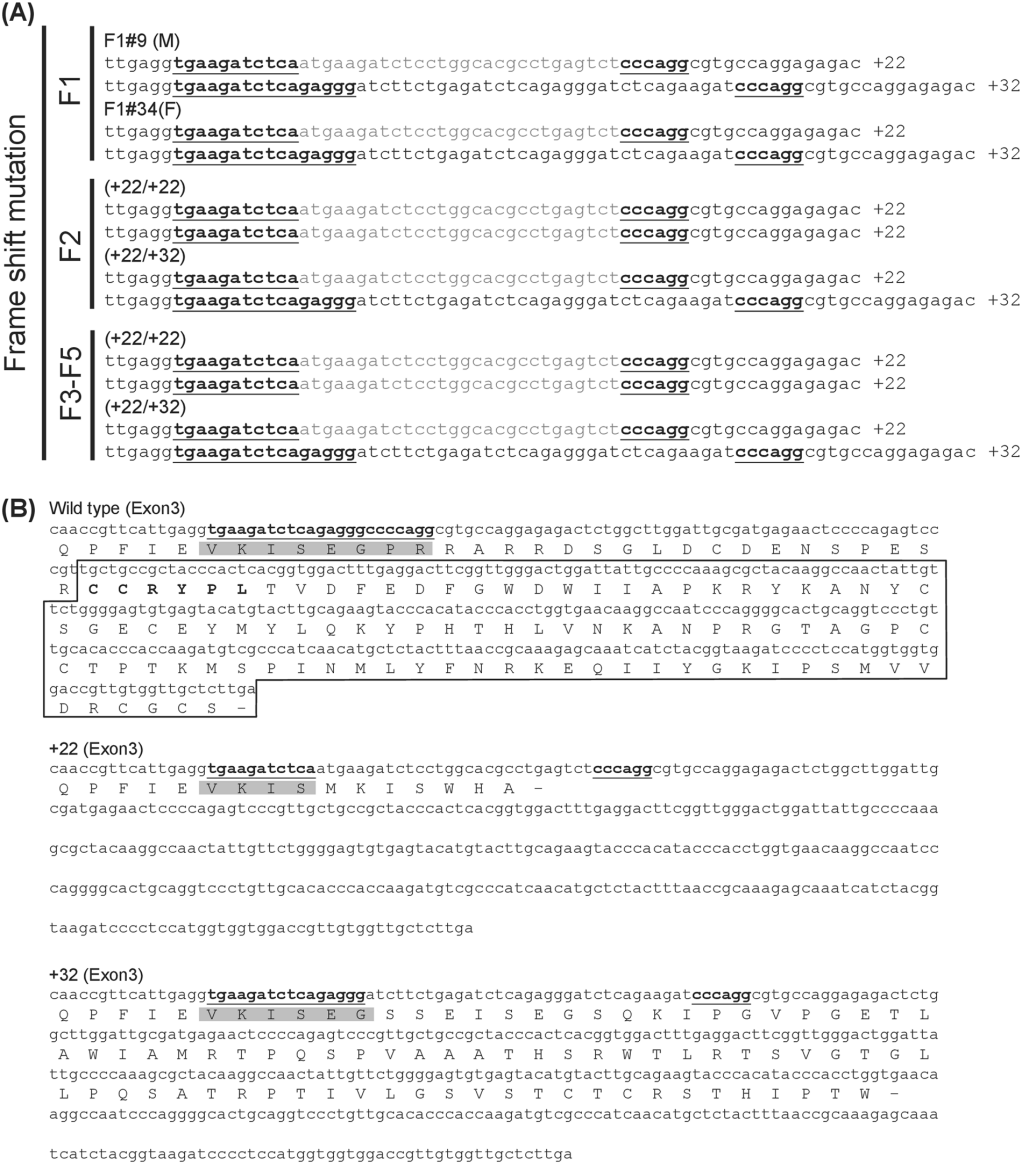
Genotypes of the two selected MSTN^−/−^ medaka mutants produced by CRISPR/Cas9-mediated genome editing. (**A**) After sequence analysis confirmed that F1#9 M and F1#34 F both harbored the same two frame-shifted MSTN mutations, these two fish were used as a breeding pair. The offspring (F2) of F1#9 M x F1#34 F presented as one of two genotypes, both of which had CRISPR/Cas9-mediated frame-shifted MSTN mutation in both alleles. Randomly selected pairs were used to produce subsequent generations (F3-F5), all of which consisted entirely of the same two genotypes. The MSTN-sgRNA target sequence is underlined while the gray letters indicate the inserted nucleotides. (**B**) Corresponding amino acid sequences of exon 3 of the WT and two mutant genotypes. The targeted amino acids are shaded and the active C-terminal domain is boxed. The stop codon is represented by “−”.

Two F1 fish (F1#9 M and F1#34 F) with the same heterozygous mutation consisting of a 22-bp insertion and a 32-bp insertion (Fig. 2A) were crossed with each other to generate the next generation of MSTN^−/−^ medaka, F2. Two MSTN frame-shifted genotypes were found in 19 tested MSTN^−/−^ medaka F2 progeny (Fig. 2A). One genotype was a homozygous frame-shift mutation with the same 22-bp insertion, while the other was a heterozygous mutation consisting of the 22-bp insertion and 32-bp insertion. Because the MSTN gene was successfully disrupted in all the tested MSTN^−/−^ medaka F2 progeny, we were able to randomly select pairs of F2 mutants to produce the F3 generation of MSTN^−/−^ medaka. Screening of the MSTN^−/−^ F3 medaka confirmed that all 6 of the tested fish carried the same two MSTN frame-shifted genotypes as the F2 fish (Fig. 2A). The F4 and F5 generations of MSTN^−/−^ medaka were obtained in a similar way.

To confirm that there were no off-target effects, we used the web tool CHOPCHOP (https://chopchop.rc.fas.harvard.edu/index.php) to predict possible off-targets for the MSTN sgRNA sequence used in this study. An off-target site was predicted at chr4:31195001 of the medaka genome (Suppl. Fig. S1A). We designed a PCR primer set to amplify this region, and performed PCR on genomic DNAs extracted from wild type (WT) and MSTN^−/−^ medaka F5 medaka (Suppl. Fig. S1B). Sequencing showed that the resulting ~430 bp amplicons from WT and MSTN^−/−^ F5 medaka were identical, suggesting that no off-target effect had occurred on chr4:31195001 (Suppl. Fig. S1C).

Figure 2B further shows the predicted amino acid sequence of the mutated proteins, from which we infer that functional MSTN was no longer being expressed in either of these mutants. These results showed that our MSTN-sgRNA CRISPR/Cas9 system successfully induced frame-shift mutations in exon 3 of the medaka MSTN genome, and that these genomic mutations were successfully transmitted to the subsequent generations. To check whether there was any frame-shift correction of the transcribed MSTN mRNA, we designed a PCR primer set to amplify the C-terminal region of the mRNAs from WT and MSTN^−/−^ F5 medaka (Suppl. Fig. S2A). The expected 22-bp insertion was detected in the C-terminus of the MSTN mRNA in each of the MSTN^−/−^ F5 medaka (Suppl. Fig. S2B). However, in two of the knockout fish (MSTN^−/−^ #5 and #8), an unexpected 61-bp deletion was sometimes detected even though the genotype of these MSTN^−/−^ F5 medaka all showed the 22-bp insertion in the C-terminus of the MSTN gene. We therefore infer that the 61-bp deletion was probably due to an RNA splicing event during MSTN mRNA maturation. Fortunately, the 61-bp deletion still caused a frame-shifted mutation, suggesting that these fish would still have expressed MSTN with a non-functional C-terminus (Suppl. Fig. S2C). We therefore conclude that expression of the functional MSTN protein in the MSTN^−/−^ F5 medaka was completely disrupted at least in all of the fish that we tested.

### Growth performance and morphology of CRISPR/Cas9-mediated MSTN^−/−^ F4 medaka

**Figure 3:**
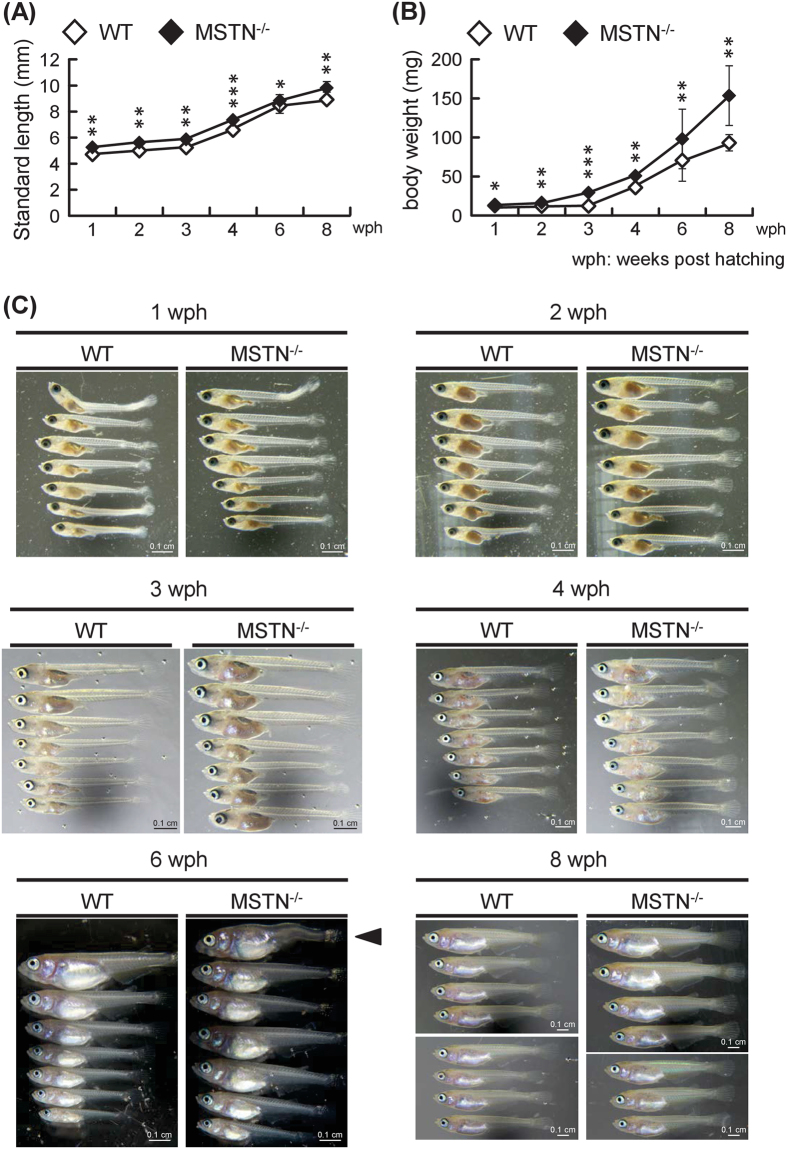
CRISPR/Cas9-mediated MSTN^−/−^ F4 medaka show better growth parameters. (**A**) Body weight and (**B**) standard length of wild type (WT) and MSTN^−/−^ F4 medaka. Values represent the mean ± SD from seven individual samples. Asterisks indicate a significant difference (*p < 0.05, **p < 0.01 and ***p < 0.001) between WT and MSTN^−/−^ F4 medaka. (**C**) Morphology of WT and MSTN^−/−^ F4 medaka from 1–8 wph. At 6 weeks post-hatching (wph), one of the MSTN^−/−^ fish had a spinal deformity (arrow head).

In general, MSTN mutations in both mammals and fish result in an increase in growth. We therefore investigated the growth performance of MSTN^−/−^ F4 medaka from 1 to 8 wph (i.e. from juvenile to adult stages) by using two indicators, the standard length and body weight^27^. As shown in Fig. 3A,B, the growth performance of MSTN^−/−^ F4 medaka was significantly better than that of WT. At 8 wph, compared with the WT group, relative body length and weight showed an increase of 10.0% and 64.9%, respectively. We also observed an accelerated increase in the body weight of the MSTN^−/−^ F4 medaka at 6 and 8 wph compared to the corresponding WT controls (Fig. 3B). Although the MSTN^−/−^ medaka exhibited a longer and wider body trunk than the WT controls, most of them showed relatively normal morphology (Fig. 3C). However, some of the mutants presented an observable deformity. Although the occurrence of this phenotype was not recorded in earlier generations, we observed severe spinal curvature in one of the MSTN^−/−^ F4 medaka at 6 wph (Fig. 3C) as well as in several of the adult MSTN^−/−^ F5 medaka (Suppl. Fig. S3A). To check the genotype of the three MSTN^−/−^ F5 medaka in Suppl. Fig. S3A and two other MSTN^−/−^ F5 medaka that also presented with the same spinal deformity, we used PCR followed by sequencing of the 450 bp amplicon that includes the MSTN-sgRNA target site. All of the tested deformed fish showed a homozygous 22 bp insertion at the target site (Suppl. Fig. S3B). (Unless otherwise noted, none of the deformed MSTN^−/−^ medaka were used in subsequent parts of this study.)

### The expression of MRFs in CRISPR/Cas9-mediated MSTN^−/−^ F4 medaka

**Figure 4:**
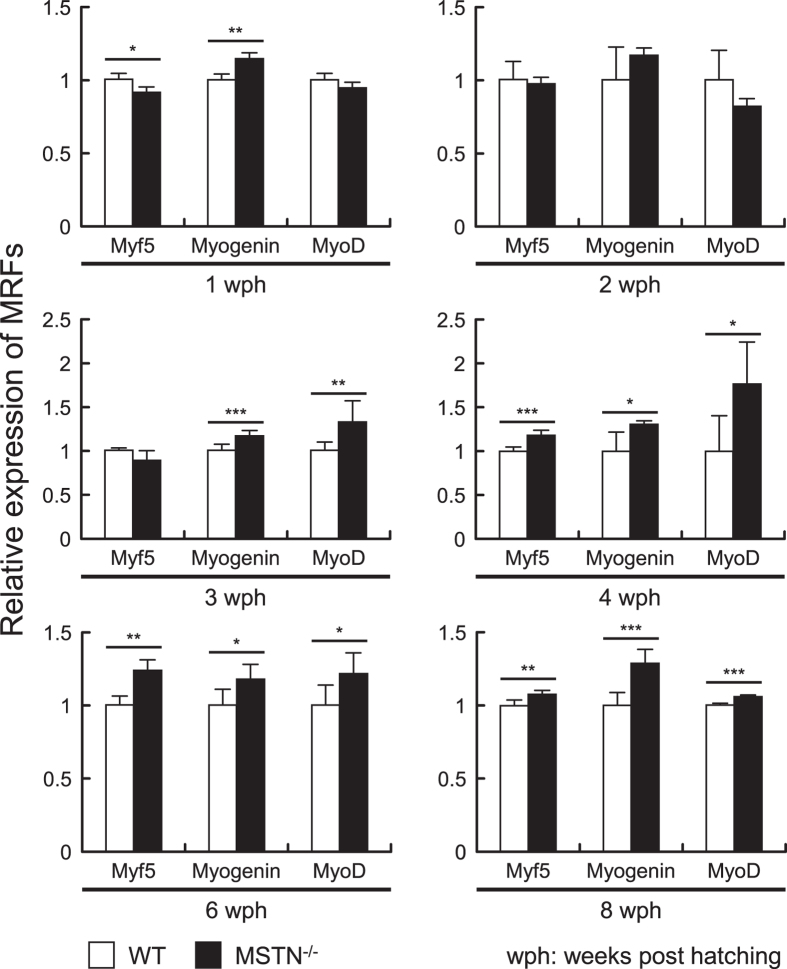
Gene expression levels of myogenic regulatory factors (MRFs) were increased in MSTN^−/−^ F4 medaka. Gene expression in each sample was analyzed by real-time PCR, and the expression levels of the MRFs were normalized against EF-1α mRNA levels. The relative expression levels are shown as fold change compared to the WT group. Each bar represents the mean ± SD from 6–7 individual samples. Asterisks indicate a significant difference (*p < 0.05, **p < 0.01 and ***p < 0.001) between WT and MSTN^−/−^ medaka.

Because MSTN is known to activate the Smad2/3 pathway, which in turn leads to the down-regulation of MRFs (myogenic related factors), we next investigated the expression of the Smad2/3-regulated MRFs in MSTN^−/−^ F4 medaka from 1 to 8 wph. mRNA was extracted from whole WT and MSTN^−/−^ F4 medaka and subjected to real-time PCR to detect the expression of three major MRFs: Myf5, Myogenin and MyoD^22, 27^. Starting from the post-juvenile stage (4 wph) the expression levels of the three tested MRFs were significantly upregulated in the MSTN^−/−^ F4 medaka (Fig. 4). Although these results do not demonstrate any direct relationship between MSTN knockout and Smad, they nevertheless suggest that within 4 weeks of hatching, the Smad 2/3 pathway may be suppressed in MSTN^−/−^ F4 medaka.

### Effect of CRISPR/Cas9-mediated MSTN mutation on skeletal muscle development in MSTN^−/−^ F4 fish

**Figure 5:**
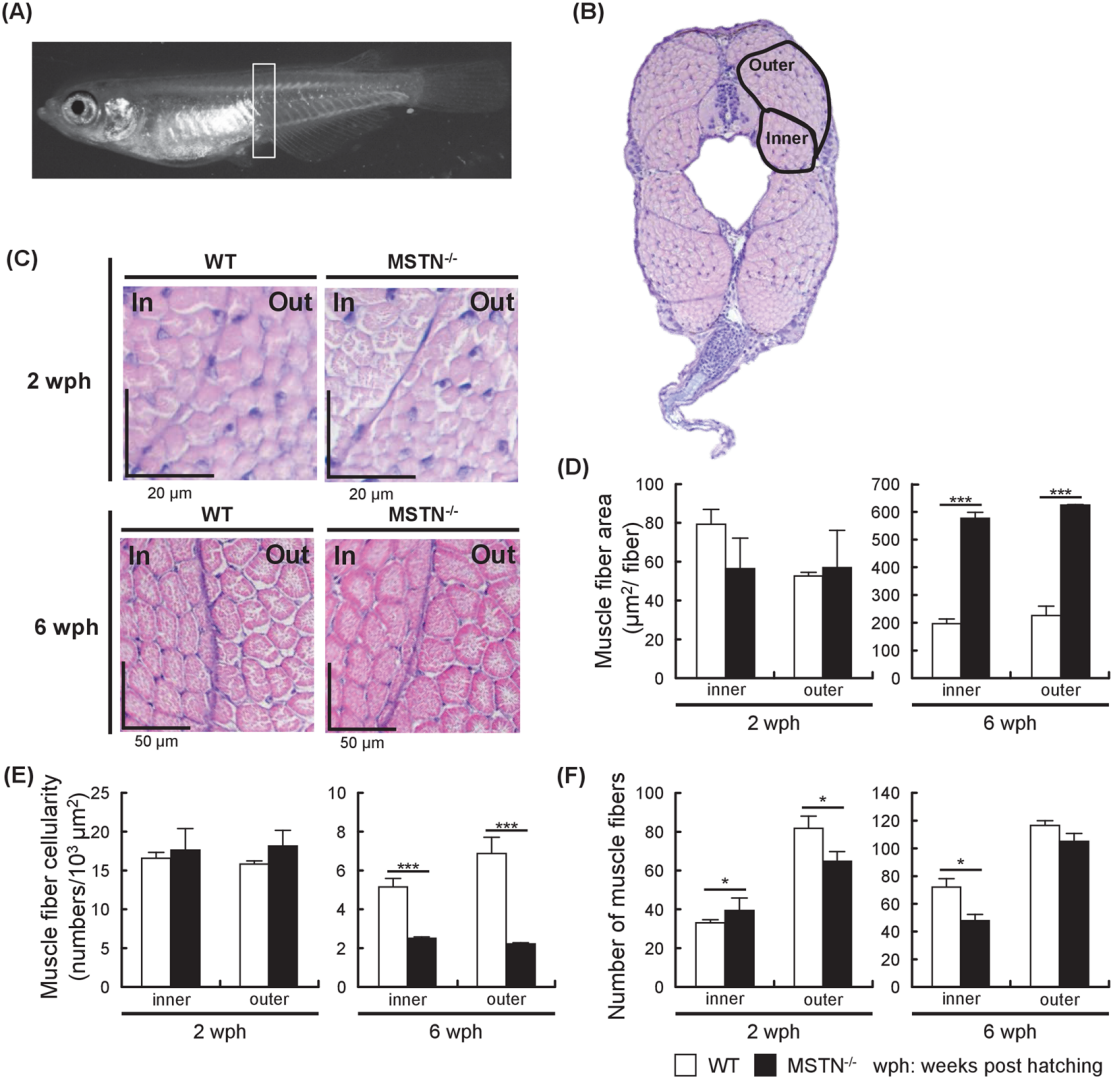
Histological sections showed evidence of hypertrophic muscle growth in MSTN^−/−^ F4 fish. (**A**) The cross-sections used in this assay were taken from the area shown in the white box. (**B**) Typical cross-section showing location of the inner and outer compartments^24^ of the epaxial muscles adjacent to the vertebral column. (**C**) H&E staining of the muscle fibers in the indicated compartments of WT and MSTN^−/−^ medaka at 2 and 6 wph. (**D**) Cross-sectional muscle fiber area in the inner and outer compartments (as measured in 10 muscle fibers in 3~4 fish). (**E**) Muscle fiber density and (**F**) total numbers of fibers (as measured in 3~4 fish). Values represent the mean ± SD. Asterisks indicate a significant difference (*p < 0.05, **p < 0.01 and ***p < 0.001) between WT and MSTN^−/−^ medaka.

To monitor skeletal muscle formation in MSTN^−/−^ F4 fish, we tracked the muscle fiber cellularity and the muscle fiber area using histological analysis. For this experiment, we focused on the inner and outer compartments of the epaxial muscles adjacent to the vertebral column as shown in Fig. 5A,B. Histological examination showed that the muscle fibers in both areas of the MSTN^−/−^ F4 medaka at 6 wph had a larger cross-sectional area compared to those in WT medaka (Fig. 5C). This observation is reflected in the quantitative data shown in Fig. 5D,E. The cross-sectional area of the average muscle fiber in MSTN^−/−^ F4 medaka at 6 wph was ~ 3 times bigger than that in WT medaka (Fig. 5D), while the muscle fiber cellularity of both the inner and outer compartments of the epaxial muscles was significantly decreased in MSTN^−/−^ F4 medaka at 6 wph (Fig. 5E). We further found that, even when there was a significantly different number of muscle fibers in these two compartments, the difference was relatively small (Fig. 5F). Taken together, these data suggest that CRISPR/Cas9-mediated MSTN mutation in MSTN^−/−^ F4 fish caused muscle hypertrophy, not hyperplasia.

### Effect of CRISPR/Cas9-mediated MSTN mutation on the response to virus challenge in MSTN^−/−^ F4 fish

**Figure 6:**
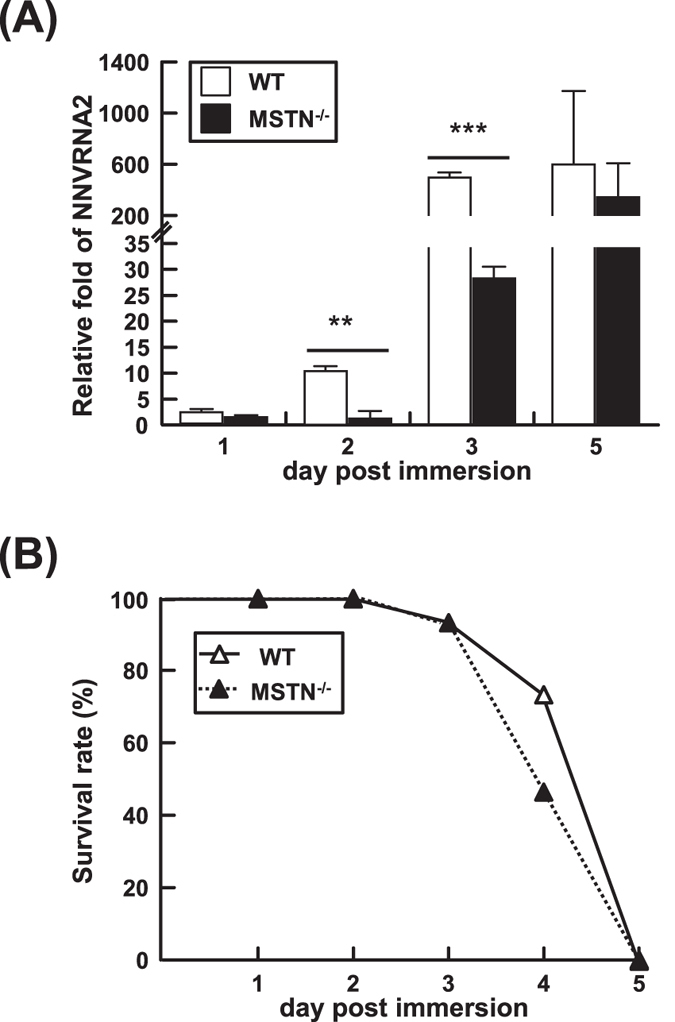
Evidence of increased virus susceptibility of MSTN^−/−^ F4 fish. (**A**) Relative expression level of RGNNV RNA2. Since RGNNV is an RNA virus, the amount of RGNNV RNA2 reflects both the number of virus genome copies plus the transcribed virus mRNA. (**B**) The survival rate after immersion challenge with RGNNV. Values represent the mean ± SD. Asterisks indicate a significant difference (*p < 0.05, **p < 0.01 and ***p < 0.001) between WT and MSTN^−/−^ medaka.

There is a well-known trade-off between growth and immunity that has been reported in a range of animals. To assess this effort in medaka, MSTN^−/−^ F4 fish were challenged with RGNNV by immersion at 1 wph. RGNNV RNA2 expression was used as an indicator of the infection state (Fig. 6A), and the survival rate was also recorded (Fig. 6B). While the survival rates were almost identical in both groups, surprisingly, at 2~3 days post immersion, the expression of RGNNV RNA2 was significantly lower in MSTN^−/−^ fish. Although the reason for the low virus copy number is unknown, these results suggest that MSTN^−/−^ fish are sensitive to RGNNV infection even when the copy number is low.

### Effect of CRISPR/Cas9-mediated MSTN mutation on reproductive performance of MSTN^−/−^ F4 medaka

**Figure 7:**
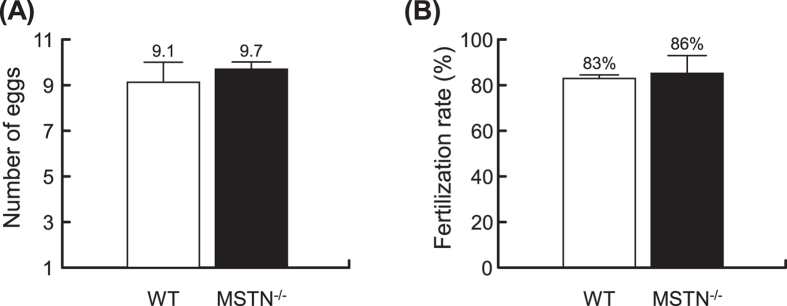
Egg production and fertilization rate of MSTN^−/−^ F4 medaka were not impaired. (**A**) The number of eggs and (**B**) fertilization rates were monitored in females of breeding pairs in WT and MSTN^−/−^ F4 medaka at 20 sampling time points during 2 months. Values represent the mean ± SD from four pairs of WT and three pairs of large phenotype/non-deformed MSTN^−/−^ F4 medaka. Asterisks indicate a significant difference (*p < 0.05, **p < 0.01 and ***p < 0.001) between WT and MSTN^−/−^ medaka.

To assess reproductive performance of MSTN^−/−^ F4 fish, we investigated the egg numbers and fertilization rate of 3~4 pairs of WT and large phenotype/non-deformed MSTN^−/−^ F4 medaka. During two months of observation, mean egg numbers were 9.1 and 9.7 in the samples of WT and MSTN^−/−^ F4 medaka, while the fertilization rates were 83% and 86%, respectively (Fig. 7). There was no significant difference in these two reproductive indicators between the WT and MSTN^−/−^ F4 medaka. We conclude that CRISPR/Cas9-mediated MSTN mutation does not significantly affect the reproductive performance of MSTN^−/−^ F4 medaka.

## Discussion

Although the CRISPR/Cas9 system is now widely used to edit target genes in various mammals^22, 23, 34–37^ and in zebrafish, it is still only rarely applied to other fish species. Even in medaka, which is another fish model animal, to date, there has only been one published study. This recent study was the first to use CRISPR/Cas9 to successfully target the medaka Dj-1 gene, and it also showed how the resultant mutation in the founder (F0) generation could subsequently be transmitted to the F1 genotypes^38^. Here in the present study, by using the CRISPR/Cas9 system, we not only document the mutated MSTN genotypes of the MSTN^−/−^ medaka, we also continued to observe the specific phenotypes through to the F4 and F5 generations. Although other platforms have been used to generate MSTN-mutated fish, to our knowledge, this is the first investigation of the MSTN^−/−^ phenotypes produced by the CRISPR/Cas9 system in medaka, and also the first to document the MSTN^−/−^ fish for several generations.

Myostatin is a key suppressor of skeletal muscle growth that prevents overdevelopment via the activation of the Smad2/3 pathway to decrease the expression of MRFs, and as expected, in the MSTN^−/−^ F4 medaka, three major MRFs were upregulated from 4 weeks post hatching (Fig. 4). Similar results were found for the same three MRFs (MyoD, Myf5 and Myogenin) in TALENs-mediated MSTN^−/−^ F2 medaka^22^. Like most MSTN-deficient terrestrial vertebrates and fishes^27, 39^, CRISPR/Cas9-mediated MSTN^−/−^ medaka also showed a phenotype with better growth performance (Fig. 3), with the acceleration of body weight being proportionally greater than the increase in body length. This body shape, ie the thicker body trunk, is consistent with the phenotypical changes that are seen in transgenic zebrafish that express MSTN antisense RNA^21^, MSTN C315Y mutant medaka produced by the TILLING method^27^ and TALENs-mediated MSTN^−/−^ medaka^22^. Clearly, all of these results suggest that MSTN deficiency in medaka results in an increase of the growth rate. We further note that this is true even though all the medaka in the TALENs study, both WT and MSTN^−/−^, had a phenotype with a much longer body than the fish used in the present study (eg ~17 mm and ~90 mg at 8 wph for WT in the TALENs study, versus ~10 mm and ~95 mg at 8 wph in the present study).

Muscle mass depends on both the number and size of the muscle fibers, and myostatin appears to be able to act on both^40^. Thus when down-regulation or mutation of MSTN leads to an increased growth rate in most mammals and various fish species, this can be due to either hypertrophy and/or hyperplasia. However, the observed effects are not always consistent even within a species, with different platforms sometimes producing opposite results. For example, mice with a MSTN C313Y missense mutation showed hyperplasia without hypertrophy^16^, while MSTN knockout mice in which the C-terminal region was replaced by a neo cassette had an increased muscle mass that was due to both hypertrophy and hyperplasia^1^. This difference cannot simply be due to incomplete vs complete removal of MSTN functionality by the two respective platforms, because another study reported that transgenic mice that expressed a dominant negative MSTN also showed hypertrophy with hyperplasia^41^. Similarly inconsistent phenotypes have also been found in zebrafish. Zebrafish that expressed a dominant negative MSTN showed hyperplasia without hypertrophy^23^, whereas transgenic zebrafish that expressed MSTN antisense RNA as well as zebrafish in which MSTN had been transiently inactivated by oral administration of MSTN D76A dominant negative recombinant protein both presented with hypertrophy only^21, 42^. In the present study, our use of the CRISPR/Cas9 platform to disrupt the bioactive C-terminal domain in exon 3 of the medaka MSTN genes (Figs 1 and 2; Suppl. Fig. S2) would have completely prevented expression of the functional MSTN active domain. As noted above, histological analysis showed that while the mean diameter of the muscle fibers in MSTN^−/−^ F4 fish was 2.7~2.9 fold larger than in WT (Fig. 5D,E), there was no increase in the number of muscle fibers (Fig. 5F). These results suggest that hypertrophy, not hyperplasia, is the prime mechanism of the additional muscle growth in CRISPR/Cas9-mediated MSTN^−/−^ medaka. By contrast, in MSTN C315Y mutant medaka produced by the TILLING method, hypertrophy became significant only at the late adult stage (16 wph) while *mstnC315Y*-induced hyperplasia was seen from the post-juvenile stage onward^27^. Further, in 6-month-old transgenic medaka in which the dominant-negative form of myostatin was overexpressed, the cross-sectional area of each muscle fiber was actually reduced while the number of fibers increased by a factor of between 1.8~2.4 ^24^. It will probably not be easy to tease apart the extent to which these results are due to the different platforms. Nevertheless, it seems likely that the presence of endogenous full-length MSTN, even with a TILLING-induced in-frame point mutation, might well lead to different phenotypes than those seen in the off-frame mutations produced by CRISPR/Cas9. To explore these possibilities in detail, more work will need to be done.

Even though most of the CRISPR/Cas9-mediated MSTN^−/−^ medaka had an acceptable, relatively normal body shape with a thicker body trunk, some of them (in one batch of 60 eggs, as many as 7 individuals [~12%]) had a spinal deformity (Figs 3C and S3A). This phenotype may have been due to disruption of bone formation, and curiously, we note that MSTN has previously been shown to regulate bone formation in mice: in MSTN knockout mice, total bone cross-sectional area, callus strength and bone mineral density were all increased^43^, and some tendons also become smaller and less flexible^44^. Nevertheless, at this time, the underlying reason for these spinal deformities is still unknown, and in particular the extent to which myostatin may have been involved remains unclear.

It is a fundamental concept that in the course of an organism’s life history, it needs to allocate resources between competing physiological processes and attempt to optimize the trade-off between various fitness-enhancing traits. One commonly observed trade-off in fish is growth versus reproduction^45–47^. However in the present study, we found that the reproductive ability of the large phenotype CRISPR/Cas9-mediated MSTN^−/−^ medaka was not affected (Fig. 7). Another well-known example is the trade-off between growth and immunity. This kind of trade-off has been studied in poultry^32^, bull calves^48^ and sticklebacks^49^. In a previous study, we also found that TALENs-mediated MSTN^−/−^ medaka had both a larger phenotype and a compromised immune system^22^. In those fish, there was a down-regulation of immune genes, significantly higher accumulation of virus, and an increased susceptibility to infection by RGNNV. By contrast, when we challenged the CRISPR/Cas9-mediated MSTN^−/−^ medaka with the same virus, we found that the virus copy numbers were even lower than in WT (Fig. 6A). The reason for this discrepancy is not clear, but it may be related to the different phenotypes used in the two studies; as noted above, the fish used here were ~40% shorter than those in the TALENs study. Although it is difficult to directly compare results across these two studies, in the present case, by diverting more resources toward growth, the medaka might reduce the availability of these resources to the virus – and thus limit the virus’s ability to replicate. Nevertheless, despite the reduced viral load, the mortality rate of the MSTN^−/−^ F4 medaka was almost identical to that of the WT (Fig. 6B), suggesting that the immune system in these fish must somehow be impaired and that their susceptibility was greater. This further suggests that while both TALENs and CRISPR/Cas9 systems induce similar muscle growth in MSTN^−/−^ fish, at the same time, they also compromise the immune system in different ways. If so, a side-by-side comparison of these two platforms might provide some very useful insights into the underlying mechanisms of immune suppression in MSTN^−/−^ medaka.

## Materials and Methods

### Experimental fish and ethics statement

The medaka (*Oryzias latipes*; Cab strain) used in this study were maintained in transparent plastic rearing containers with a recirculating water system at 28 °C, and a 14/10-h day/night cycle. The fish were fed three times a day with freshly hatched *Artemia* nauplii or commercial feed. All animal experiments in this study were approved by the Laboratory Animal Center of National Cheng Kung University and carried out in accordance with approved guidelines.

### Vectors used for Cas9 nuclease and sgRNA expression

The two plasmids used for the CRISPR/Cas9 system in this study were obtained from Addgene: pCS2 + hSpCas9 (#51815) and pDR274 (#42250). The pCS2 + hSpCas9 vector is the Cas9 expression vector with a SP6 promoter for Cas9 nuclease expression, while the pDR274 vector has a T7 promoter and was used for sgRNA expression.

### sgRNA design and cloning into the expression vector

The genomic sequence of medaka *mstn* (*Ol*MSTN) was obtained from the medaka genome database at the Ensembl Genome Database Project (Ensembl gene no. ENSORLG00000015057). Design and construction of *Ol*MSTN-sgRNA followed a previously described protocol^38, 50^. In brief, the selected target site of the *Ol*MSTN-sgRNA was a sequence of the form 5′- N_21_GG -3′, as recommended in a design guideline published by Ran *et al*.^31^. A pair of the selected *Ol*MSTN-sgRNA oligonucleotides in the sense and antisense directions were synthesized by Mission Biotech Co., Ltd. (Taiwan) using an oligonucleotide purification cartridge (OPC). The *Ol*MSTN sgRNA was then cloned into the pDR274 vector using the protocols described in Ansai and Kinoshita^38^. The sequences of the *Ol*MSTN-sgRNA oligonucleotides are listed in Table 1.

**Table 1:** MSTN-sgRNA and PCR primers used in this study.

	Name	Sequence (5′-3′)	Usage	Reference
MSTN-gRNA	*Ol*MSTN-Sense*	*TAGG*AAGATCTCAGAGGGCCCC	CRISPR/Cas9 target site	This study
	*Ol*MSTN- Antisense*	*AAAC*GGGGCCCTCTGAGATCTT		
Primers	*Ol*MSTN_F2	TCAAGTGCTCACTCAGGCTG	PCR for *Ol*MSTN genotype	ENSORLG00000015057
	*Ol*MSTN_R3	CCACCGTGAGTGGGTAGC		
	*Ol*MyoD-qPCR-F	GCCCCCGCTCCAACTG	qPCR	Chiang *et al*.^22^
	*Ol*MyoD-qPCR-R	CGTCTGACACCTCGGTCCAT		
	*Ol*Myf5-qPCR-F	CGGCGGCTCAAAAAGGT	qPCR	Chiang *et al*.^22^
	OlMyf5-qPCR-R	GAGGTGCAGCGCCTCAGT		
	*Ol*Myogenin-qPCR-F	TTGCCCACCATGGAGCTT	qPCR	Chiang *et al*.^22^
	*Ol*Myogenin-qPCR-R	AGCGCTGGTCAGGAAAGAAG		
	NNV_RNA2_qPCR_F	5′- GACGCGCTTCAAGCAACTC -3′	qPCR	Chiang *et al*.^22^
	NNV_RNA2_qPCR_R	5′- CGAACACTCCAGCGACACAGTA -3′		
	*Ol*EF-1α-qPCR-F	ATTTGCGGGGTTTGCAC	qPCR	Chiang *et al*.^22^
	*Ol*EF-1α-qPCR-R	TGGGACTTTTATACGGACTGG		
	Anchor-dTv	GACCACGCGTATCGATGTCGACTTTTTTTTTTTTTTTTV	cDNA synthesis	

*Cutting sites are italicized.

### Synthesis of Cas9 mRNA and *Ol*MSTN-sgRNA

After pCS2 + hSpCas9 was digested by *Not*I treatment, this linearized vector was used as the template for synthesizing capped Cas9 mRNA with a mMessage mMachine SP6 Kit (Life Technologies). The resulting Cas9 mRNA was purified by using a RNeasy Mini Kit (Qiagen). For synthesis of *Ol*MSTN-sgRNA, the pDR274 vector containing *Ol*MSTN-sgRNA was first digested by *Dra*I and then used as the template for sgRNA synthesis using an AmpliScribe T7-Flash Transcription Kit (Epicentre). Ammonium acetate precipitation was used to purify the synthesized *Ol*MSTN-sgRNA.

### Embryo microinjection of Cas9 mRNA and *Ol*MSTN-sgRNA

Microinjection of the medaka embryos followed a method described previously by Kinoshita *et al*.^51^. In brief, fertilized eggs were collected within 30 min after spawning and kept in pre-chilled Iwamatsu’s balanced salt solution^52^ at 4 °C to arrest development. A mixture containing 100 ng/µL of Cas9 mRNA and 25 ng/µL of *Ol*MSTN-sgRNA was prepared and injected into the fertilized eggs at the one-cell stage. The injected embryos were then incubated at room temperature in medaka embryo culture medium (0.0001% methylene blue, 0.1% NaCl, 0.3% KCl, 0.004% CaCl_2_. 2H_2_O, 0.016% MgSo_4_. 7H_2_O). After hatching, the larvae were raised to sexual maturity and used as “founder” fish (F0).

### Detection of *Ol*MSTN-CRISPR/Cas9 mediated DNA mutations in F0 founder fish and their offspring

To observe the genomic DNA mutations induced by Cas9 and *Ol*MSTN-sgRNA in CRISPR/Cas9-mediated MSTN-mutated medaka, a small piece of the caudal fin from individual F0 fish was collected and subjected to genomic DNA analysis as previously described^22, 38^. The primer set *Ol*MSTN F2/*Ol*MSTN R3 (Table 1) was used to amplify the region containing the *Ol*MSTN-sgRNA target site in the genomic DNAs extracted from wild type (WT; non-genome-edited Cab strain) and CRISPR/Cas9 treated medaka. The resulting amplicons were then analyzed by heteroduplex mobility assay (HMA) as described previously^53^. The amplicons were also cloned into TA cloning vector (Bioman, Taiwan) and the mutations were sequenced. After the above screening had confirmed the occurrence of CRISPR/Cas9-mediated MSTN mutation in the F0 generation, these founder fish were crossed with each other, and their offspring (F1) were checked for MSTN mutations in the same way.

### Breeding of the F2-F5 MSTN^−/−^ generations

Two of the F1 MSTN^−/−^ progeny with the same frame-shifted mutation patterns, F1#9(M) and F1#34(F), were mated to produce the F2 generation. The F2 generation were crossed with each other to produce F3 progeny, and the F4 and F5 progeny were subsequently bred in the same way. The F2 and F3 generations were screened as described above to confirm that the same mutation patterns were successfully inherited. Since F3 was the first generation to produce enough fish for the following assays, only F4 fish were used for this study.

### Collection and assessment of WT and MSTN^−/−^ F4 medaka

WT and F4 eggs were collected daily, and each cohort of larvae (~30) from eggs collected on the same day were maintained in a separate plastic rearing container (3 L) under a 14/10-h day/night cycle at 26 °C. For each group of WT and MSTN^−/−^ F4 fish, at least 7 tanks were used for growth assessment, quantification of the expression of MRFs (myogenic related factors) genes expression, and histological analysis. At 1, 2, 3, 4, 6 and 8 weeks post-hatching (wph), 7 individual fish were collected for growth assessment and quantification of gene expression. At 2 and 6 weeks post-hatching, another 7 individual fish were collected and fixed for histological analysis using Davidson’s fixative solution (33% EtOH, 22% formalin, and 11.5% acetic acid).

### Growth assessment of WT and MSTN^−/−^ F4 medaka

To determine the growth phenotype of WT and CRISPR/Cas9-mediated MSTN^−/−^ F4 medaka, the body weight and standard length were measured using a protocol described previously^22^. Briefly, after fish were anesthetized in 0.003% eugenol (Sigma-Aldrich, Inc., MO, USA), the fish were weighed, and after being photographed under a digital microscope, their standard length was measured. The same fish were then immediately transferred to a 1.5 ml tube containing RNA keeper (Protech Technology Enterprise Co., LTD) and subjected to RNA extraction.

### Quantification of the mRNA expression of myogenic related factors (MRFs) in WT and MSTN^−/−^ F4 medaka using real-time PCR

For this experiment, 7 individual WT and MSTN^−/−^ F4 medaka were collected at 1, 2, 3, 4, 6 and 8 weeks post-hatching as described above. After RNA extraction, total cDNA was synthesized from each sample using Superscriptase II Reverse Transcriptase (Invitrogen) with Anchor-dTv primer (Table 1). To quantify the relative expression of three major MRFs (MyoD, Myf5 and Myogenin) and the normalization factor EF-1α, real-time PCR was performed with the specific primer sets listed in Table 1. Data values were calculated by the 2^−ΔΔCT^ method and the expression level of each gene in the WT group was set to 1. Statistically significant differences between WT and MSTN^−/−^ F4 medaka were analyzed by Student’s t-test.

### Histological analysis of skeletal muscle and muscle fiber density in WT and *Ol*MSTN-CRISPR/Cas9 mediated MSTN^−/−^ F4 medaka

WT and MSTN^−/−^ medaka were fixed with Davidson’s fixative solution and decalcified using EDTA solution (400 mM EDTA, 50 mM Tris-HCl, pH8.3) as described by Chisada *et al*.^27^. Before routine dehydration and paraffin embedding, the fixed fish were pre-embedded in agar-gelatin mixture (1% agar dissolved in 2.5% gelatin solution [300 Bloom])^54^. Paraffin sections (4 μm) were mounted on glass slides, dewaxed and dehydrated, and then stained with hematoxylin and eosin. Muscle formation was measured following the protocol described by Sawatari *et al*.^24^ and Chisada *et al*.^27^. Briefly, cross-sections of all specimens were made at the anterior end of the anal fin in the anteroposterior direction. In the inner and outer compartments of the epaxial muscles adjacent to the vertebral column, we used ImageJ software (http://rsb.info.nih.gov/ij/) to count the total number of fibers, muscle fiber cellularity (i.e. density of muscle fibers), and the cross-sectional muscle fiber area in 3~5 fish from each group. Statistically significant differences between WT and MSTN^−/−^ F4 medaka were analyzed by Student’s t-test.

### The response to virus challenge in WT and *Ol*MSTN-CRISPR/Cas9 mediated MSTN^−/−^ F4 medaka

Medaka at 1 week after hatching were challenged with RGNNV (red spotted grouper nervous necrosis virus) by bath immersion following the method described by Furusawa *et al*.^55^ and Chiang *et al*.^22^. Briefly, fish were challenged RGNNV at 10^9^ TCID_50_ RGNNV/L for 2 h and then transferred to tanks containing ultraviolet-treated water and 5 μg/ml kanamycin. The survival rate was monitored daily in two groups of 15 fish each. Two more groups of ~20 fish were used to assess the virus load by monitoring the gene expression of the coat protein encoded by RGNNV RNA2 (AY44705). Total RNAs were extracted from three fish from these groups at 1, 2, 3 and 5 days post immersion (dpi). cDNA synthesis was performed by using Superscriptase II Reverse Transcriptase (Invitrogen) and Random hexamer primer. Real-time PCR was then used to measure the expression of RGNNV RNA2 with the primer set NNV_RNA2_qPCR_F/ NNV_RNA2_qPCR_R (Table 1). The expression of RGNNV RNA2 was normalized with EF-1α by the 2^−ΔΔCT^ method and the expression level of RGNNV RNA2 at 0 dpi in the WT group was set to 1. Statistically significant differences between WT and MSTN^−/−^ F4 medaka were analyzed by Student’s t-test.

### Reproductive performance of WT and *Ol*MSTN-CRISPR/Cas9 mediated MSTN^−/−^ F4 medaka

To determine the reproductive performance of MSTN^−/−^ F4 medaka, three pairs of WT and MSTN^−/−^ F4 medaka were selected at random. Eggs from the female of each breeding pair were collected every 2~3 days for 2 months to give a total of 20 samples. For each sample, we recorded both the number of eggs produced and the number of successfully fertilized eggs as observed under a light microscope. Statistically significant differences between WT and MSTN^−/−^ F4 medaka were analyzed by Student’s t-test.

## Electronic supplementary material

Supplementary Information
