# Supplementary material for: Using CRISPR/Cas9-mediated gene editing to further explore growth and trade-off effects in myostatin-mutated F4 medaka (Oryzias latipes)
Source: Sci Rep. 2017 Sep 12;7:11435. doi: 10.1038/s41598-017-09966-9 (PMC5595883; doi:10.1038/s41598-017-09966-9)
Supplement: Supplementary file 1 — Supplementary Information [file 41598_2017_9966_MOESM1_ESM.pdf]

Supplementary information

Running title: CRISPR/Cas9-mediated MSTN$^{-/-}$ medaka

Using CRISPR/Cas9-mediated gene editing to further explore growth and trade-off effects in myostatin-mutated F4 medaka (*Oryzias latipes*)

Ying-Chun Yeh[1], Masato Kinoshita[2], Tze Hann Ng[1], Yu-Hsuan Chang[1], Shun Maekawa[1], Yi-An Chiang[1], Takashi Aoki[1], Han-Ching Wang[1,3*]

[1] Institute of Biotechnology, College of Bioscience and Biotechnology, National Cheng Kung University, Tainan 701, Taiwan.

[2] Division of Applied Bioscience, Graduate School of Agriculture, Kyoto University, Kyoto 606-8502, Japan.

3 Department of Biotechnology and Bioindustry Sciences, National Cheng Kung University, Tainan, Taiwan

Keywords: myostatin, medaka, CRISPR/Cas9, muscle hypertrophy, spinal deformity, trade off effect

***Corresponding author: Han-Ching Wang**

E-mail address: wanghc@mail.ncku.edu.tw

Phone number: +886-6-2757575 ext 65603-810

Fax number: +886-6-276-6505

Address: Institute of Biotechnology, College of Bioscience and Biotechnology, National Cheng Kung University, Tainan 701, Taiwan

Figure legends

Figure S1. No off-target effect was found in MSTN$^{-/-}$ F5 medaka. (A) A search using the online tool CHOPCHOP (https://chopchop.rc.fas.harvard.edu) identified 2 possible binding target of the MSTN sgRNA used in this study. The gene sequence chr21: 18493824 was from our gene of interest, ie MSTN, while the gene sequence chr4:31195001 was a possible off-target site with 3 mismatching base pairs. (B) A primer set, chr4:31195001F and chr4:31195001R (arrows), was designed to amplify the possible off-target site on chr4:31195001 (boxed). (C) Off-target chr4:31195001 PCR amplicons were subcloned and sequenced. Sequencing data showed that no insertion or deletion occurred on chr4:31195001 in any of the tested MSTN$^{-/-}$ F5 medaka.

Figure S2. 22-bp insertions and 61-bp deletions were found in MSTN mRNA from MSTN$^{-/-}$ F5 medaka. (A) A primer set, MSTN-Q-F and MSTN-R3 (arrows), was designed to amplify a MSTN cDNA fragment that included the MSTN-sgRNA target site (boxed). (B) The resulting PCR amplicons were subcloned and sequenced. In all of the tested fish, sequencing data showed the expected 22-bp insertion in the C-terminus of the MSTN mRNA of the knockout fish. However, in two of the knockout medaka (MSTN$^{-/-}$ #5 and #8), an unexpected 61-bp deletion was also sometimes detected even

though the genotypes of these CRISPR/Cas9 treated medaka all showed the 22-bp insertion in the C-terminus of MSTN. (C) Corresponding amino acid sequences translated from the PCR amplicons of the WT and two mutant types (22-bp insertion and 61-bp deletion) showed that both mutant types caused a frame-shifted mutation of MSTN.

Figure S3. Spinal deformity in MSTN$^{-/-}$ F5 medaka. (A) Severe spinal curvature was observed in several adult MSTN$^{-/-}$ F5 medaka. (B) PCR followed by sequencing of the 450 bp amplicon that included the MSTN-sgRNA target site (underlined) was used to check the genotype of the three MSTN$^{-/-}$ F5 medaka in Suppl. Fig. S3A and two other MSTN$^{-/-}$ F5 medaka that also presented with the same spinal deformity. All of the tested deformed fish showed a homozygous 22 bp insertion at the target site

A

MSTN specific part of sgRNA: TGAAGATCTCAGAGGGCCCCAGG		
**Off-targets**		
**Genomic location**	**Number of mismatches**	**Sequence (including mismatches)**
chr21: 18493824	0	TGAAGATCTCAGAGGGCCCCAGG
chr4: 31195001	3	TGAAGgTCTCtGAGGGCCgCAGG

B

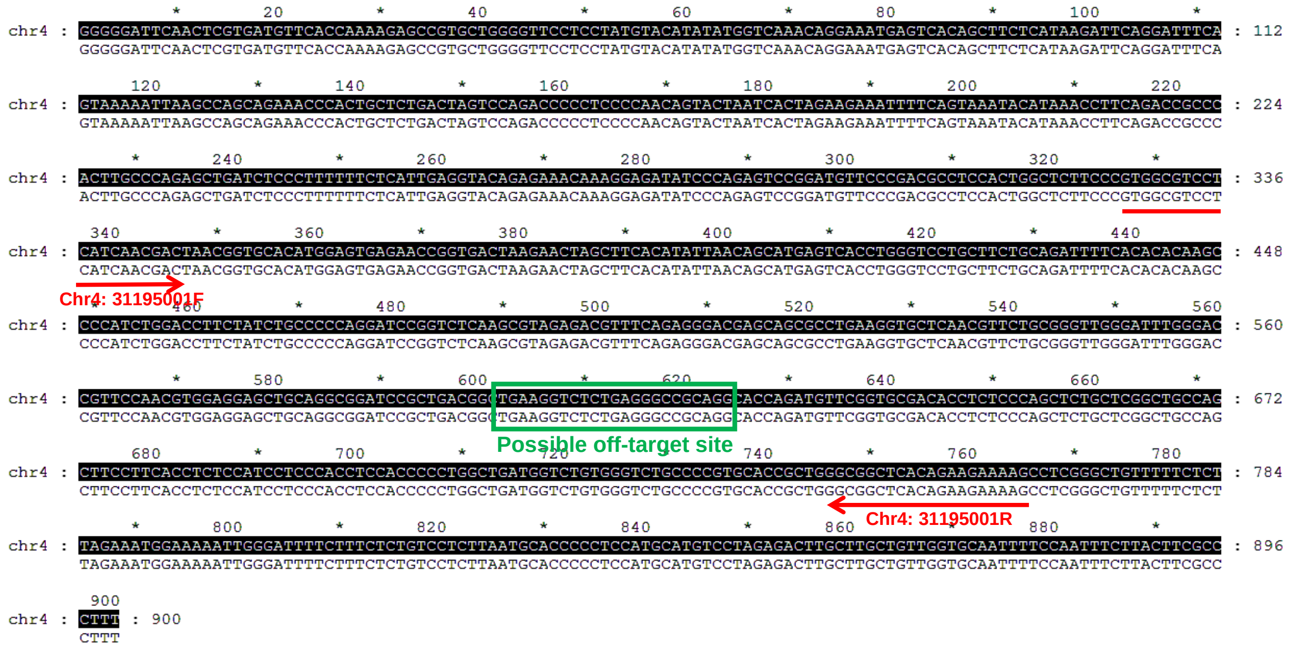

C

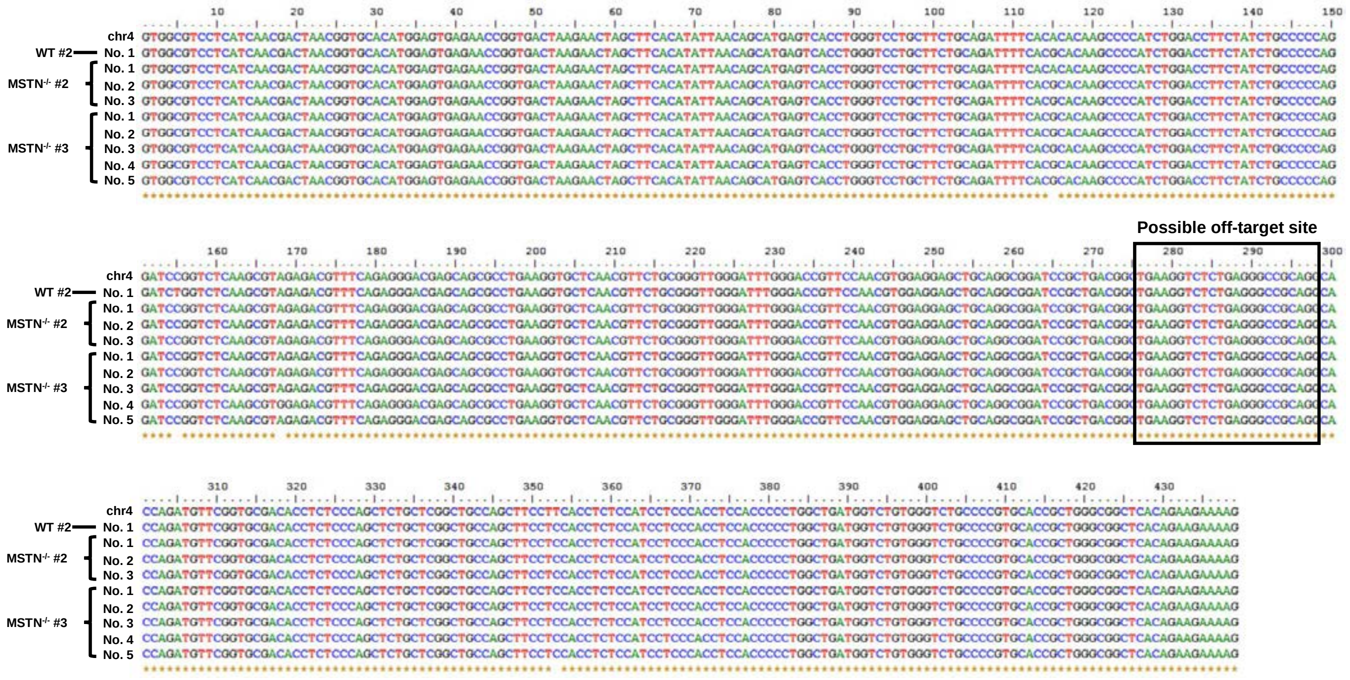

Figure S1

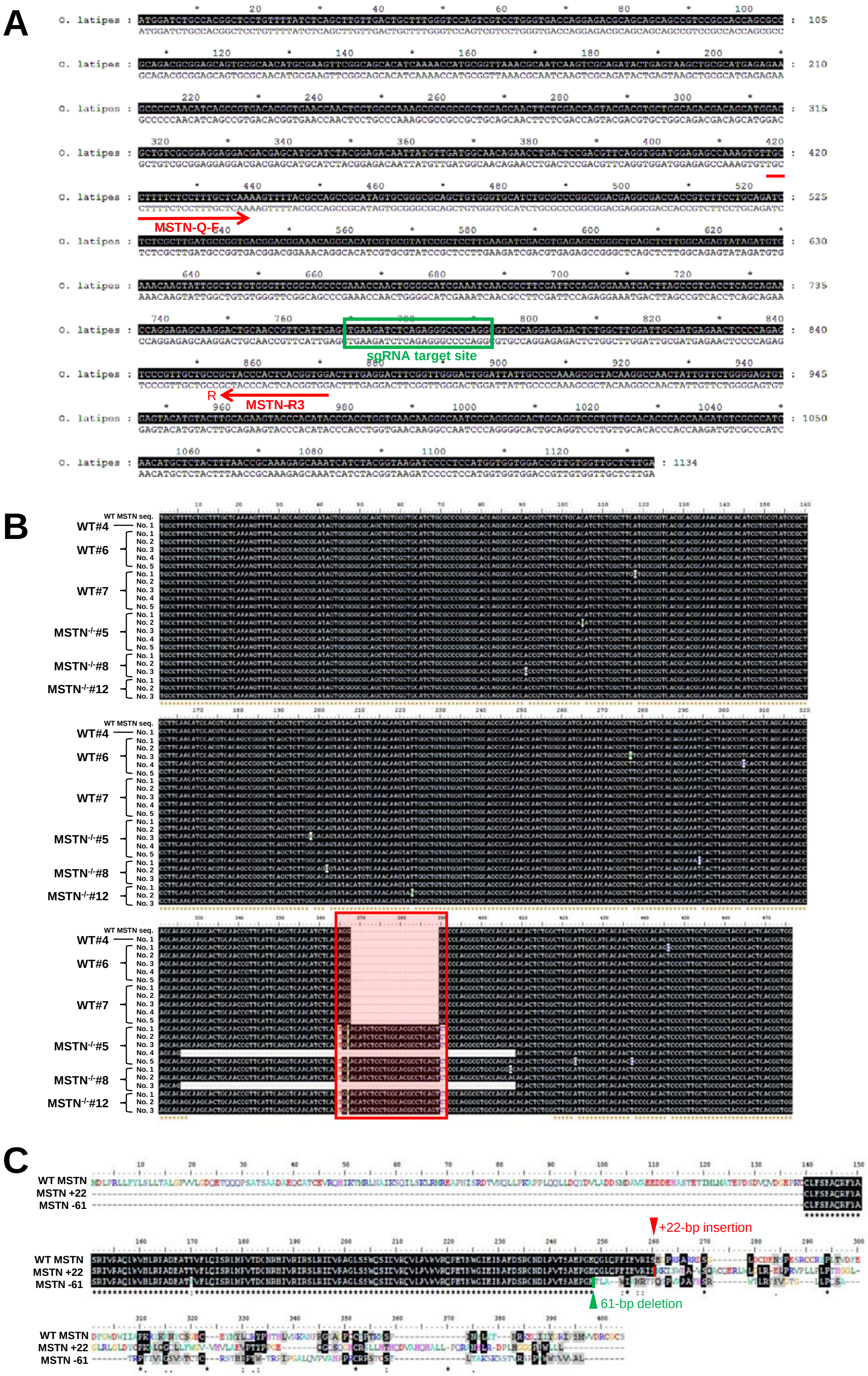
A
O. latipes
MSTN-Q-F
sgRNA target site
R
MSTN-R3
B
WT MSTN seq.
WT#4
WT#6
WT#7
MSTN-/-#5
MSTN-/-#8
MSTN-/-#12
C
WT MSTN
MSTN +22
MSTN -61
+22-bp insertion
61-bp deletion

Figure S2

A

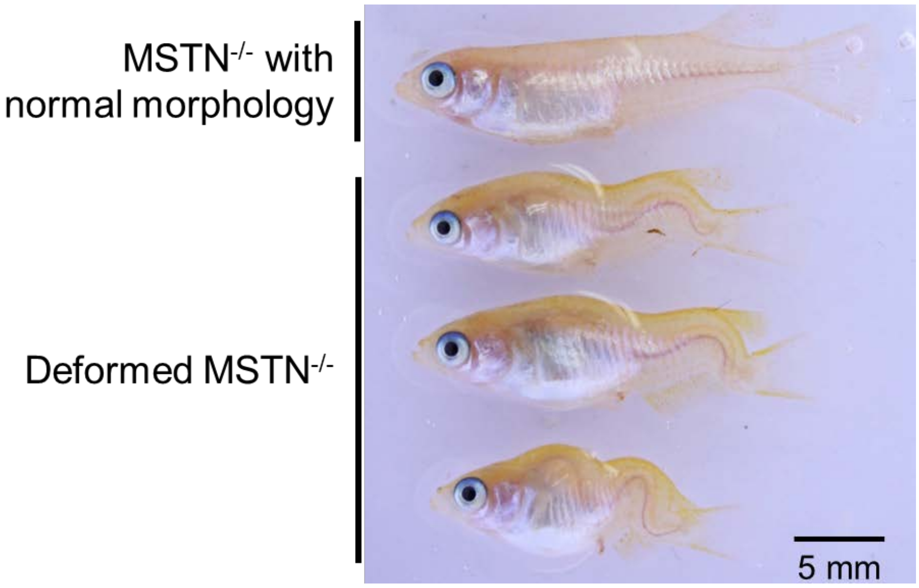

B

Deformity #1

WT TGAGGTGAAGATCTCAGAGGGCCCCAGGCGTGCCAGGAAGG

+22 TGAGGTGAAGATCTCAATGAAGATCTCCTGGCACGCCTGAGTCTCCCAGGCGTGCCA

+22 TGAGGTGAAGATCTCAATGAAGATCTCCTGGCACGCCTGAGTCTCCCAGGCGTGCCA

Deformity #2

WT TGAGGTGAAGATCTCAGAGGGCCCCAGGCGTGCCAGGAAGG

+22 TGAGGTGAAGATCTCAATGAAGATCTCCTGGCACGCCTGAGTCTCCCAGGCGTGCCA

+22 TGAGGTGAAGATCTCAATGAAGATCTCCTGGCACGCCTGAGTCTCCCAGGCGTGCCA

Deformity #3

WT TGAGGTGAAGATCTCAGAGGGCCCCAGGCGTGCCAGGAAGG

+22 TGAGGTGAAGATCTCAATGAAGATCTCCTGGCACGCCTGAGTCTCCCAGGCGTGCCA

+22 TGAGGTGAAGATCTCAATGAAGATCTCCTGGCACGCCTGAGTCTCCCAGGCGTGCCA

Deformity #4

WT TGAGGTGAAGATCTCAGAGGGCCCCAGGCGTGCCAGGAAGG

+22 TGAGGTGAAGATCTCAATGAAGATCTCCTGGCACGCCTGAGTCTCCCAGGCGTGCCA

+22 TGAGGTGAAGATCTCAATGAAGATCTCCTGGCACGCCTGAGTCTCCCAGGCGTGCCA

Deformity #5

WT TGAGGTGAAGATCTCAGAGGGCCCCAGGCGTGCCAGGAAGG

+22 TGAGGTGAAGATCTCAATGAAGATCTCCTGGCACGCCTGAGTCTCCCAGGCGTGCCA

+22 TGAGGTGAAGATCTCAATGAAGATCTCCTGGCACGCCTGAGTCTCCCAGGCGTGCCA

Figure S3
